# Comparative Genomic Analyses of Copper Transporters and Cuproproteomes Reveal Evolutionary Dynamics of Copper Utilization and Its Link to Oxygen

**DOI:** 10.1371/journal.pone.0001378

**Published:** 2008-01-02

**Authors:** Perry G. Ridge, Yan Zhang, Vadim N. Gladyshev

**Affiliations:** Department of Biochemistry, University of Nebraska, Lincoln, Nebraska, United States of America; Ecole Normale Supérieure de Lyon, France

## Abstract

Copper is an essential trace element in many organisms and is utilized in all domains of life. It is often used as a cofactor of redox proteins, but is also a toxic metal ion. Intracellular copper must be carefully handled to prevent the formation of reactive oxygen species which pose a threat to DNA, lipids, and proteins. In this work, we examined patterns of copper utilization in prokaryotes by analyzing the occurrence of copper transporters and copper-containing proteins. Many organisms, including those that lack copper-dependent proteins, had copper exporters, likely to protect against copper ions that inadvertently enter the cell. We found that copper use is widespread among prokaryotes, but also identified several phyla that lack cuproproteins. This is in contrast to the use of other trace elements, such as selenium, which shows more scattered and reduced usage, yet larger selenoproteomes. Copper transporters had different patterns of occurrence than cuproproteins, suggesting that the pathways of copper utilization and copper detoxification are independent of each other. We present evidence that organisms living in oxygen-rich environments utilize copper, whereas the majority of anaerobic organisms do not. In addition, among copper users, cuproproteomes of aerobic organisms were larger than those of anaerobic organisms. Prokaryotic cuproproteomes were small and dominated by a single protein, cytochrome c oxidase. The data are consistent with the idea that proteins evolved to utilize copper following the oxygenation of the Earth.

## Introduction

All organisms are thought to require metal ion cofactors (i.e., Fe, Zn, Mg, Mn, Co, Ni and Cu) which are involved in a wide variety of cellular processes. Additionally, certain non-metal trace elements, such as selenium (Se), and iodine (I) may be utilized. Most of these elements are necessary for redox catalysis and other enzymatic reactions, for sensing and signaling, and some serve structural roles in proteins. Defects in their homeostasis have been linked to a variety of diseases [1]–[4].

Metals are particularly important for life and may have been utilized by organisms since the time life originated on Earth. For example, before the advent of oxygenic photosynthesis, the most active ecosystems were probably driven by the cycling of H_2_ and Fe^2+^ through primary production conducted by anoxygenic phototrophs [5]. Additionally, it is thought that a substrate-level phosphorylation cycle may have been an early form of metabolism – one which required both iron and molybdenum [6].

Biological metal utilization has been driven, at least in part, by availability. As the Earth has evolved its chemical environment has significantly changed, which altered availability of certain metal ions [7], [8]. For example, as environmental oxygen increased, various metal ions, such as Cu, Co, Ni, Zn, Cd, and Mo were released from their sulfide forms and became more soluble [8], making them more readily available for biological utilization. Oxygenation of the Earth also changed the redox state of some metal ions, such as Fe (Fe^2+^ to Fe^3+^), which reduced its availability [8]. Accordingly, organisms became less dependent on the metal ions that were scarce and adapted to use the available metal ions [7].

Studying metal use in organisms can provide various insights. In this study, we use the term metalloproteome, which is a subset of the proteins from a particular proteome, which bind metal ions. Improved understanding of the composition and functions of metalloproteomes in prokaryotes, combined with our knowledge of the chemical evolution of the earth, can help decipher the evolutionary relationships among organisms and improve understanding of the roles metals play in biology. Analyses of evolutionary trends in trace element utilization have been performed for several trace elements, such as Fe and Mn [9], Se [10], [11], and Ni and Co [12].

One of the widely used trace elements is copper. This metal ion is known to be a cofactor in a number of proteins. Copper is redox-active (in biological systems it can exist as either Cu^2+^ or Cu^+^ and is more reactive in its reduced state [13]) and consequently, is a highly toxic element. The challenge, then, for copper-dependent organisms is to obtain sufficient levels of this metal ion to meet their needs, while tightly controlling intracellular copper to avoid toxicity. It is likely that little free copper exists in the cytoplasm (the same is not necessarily true for the periplasm [14]), both because of its toxicity, and because it exists at such low concentrations in the cytoplasm that it is unlikely to encounter its target proteins in a reasonable amount of time without assistance [15]. Thus, it is likely that copper is delivered to target proteins by metallochaperones.

To date, 10 Cu-containing proteins (cuproproteins) have been characterized in prokaryotes, including cytochrome c oxidase (COX), NADH dehydrogenase-2 (ND2), Cu,Zn-superoxide dismutase (SOD1), nitrosocyanin, plastocyanin, Cu-containing nitrite reductase, Cu amine oxidase, particulate methane monooxygenase (pMMO), CotA, and tyrosinase. Each of these proteins is unable to substitute other metal ions for copper. Biosynthesis of cuproproteins is dependent on high-affinity uptake of the copper ion from natural environments, regulatory proteins, and other auxiliary proteins.

In prokaryotes, two chaperones [14], [16], [17] and 9 Cu-specific transporters have been reported and only one of these transporters, CtaA, has a clearly defined role as an importer [14], [18]–[20]. CtaA imports copper from the periplasm rather than extracellular space [13], [18], [19]. Another transporter, PacS, transports copper from the cytoplasm to the thylakoid and assists in copper homeostasis [18], [19]. PacS is a class of CopA [21], another transporter. CopA presents a unique challenge as multiple different copper transporters (and cuproproteins) have been named CopA. In *Escherichia coli*, CopA is an exporter, in *Enterococcus hirae* an importer (homologous to CtaA), and in *Pseudomonas* and *Xanthomonas* a multicopper oxidase [22]. Regarding CopA transporters, there were two protein subgroups: a CopA1 subfamily containing ATPases with documented Cu^+^ influx activity, and CopA2 proteins that are more ancient and include both influx and efflux transporters spanning the entire bacterial domain [21].

In recent years, the complete genomes of numerous prokaryotic organisms have become available. Using this resource, it is possible to examine the occurrence and evolution of numerous biological pathways that organisms utilize. In this study we performed a comprehensive genome-wide investigation of both copper transport systems and cuproproteins. We analyzed 450 bacterial and 35 archaeal organisms for copper utilization. Our data revealed that copper is used by most prokaryotes and that cuproproteomes are larger in organisms living in oxygen-rich environments. Our results suggest that copper use increased as the atmosphere became more oxygenic.

## Results

### Identification of Cu-utilizing prokaryotes

We examined 450 sequenced bacterial genomes and 35 sequenced archaeal genomes for copper utilization by searching for occurrence of cuproproteins (see Table 1 for a list of reference proteins). A majority of bacteria (326 of the 450 analyzed, or 72%) were found to be copper-utilizing or users (i.e., organisms that had at least one copper-dependent protein), while the remaining 124 bacteria (28%), appeared to be nonusers (i.e., organisms in which we were unable to identify even a single cuproprotein). In contrast, among archaea, most organisms appeared to be nonusers (24 archaea, or 69%), whereas 11 archaea, or 31%, were users. Overall, copper utilization was widely distributed in prokaryotes, with more than 2/3 of all organisms being dependent on this metal. Detailed distribution of copper utilization among prokaryotes is reported in Table S1.

**Table 1:** List of cuproproteins and copper transporters, chaperones, and regulators examined in the study.

#	Protein	Protein category [reference]
1	Cytochrome C Oxidase Family	Enzyme [18]
2	NADH dehydrogenase-2	Enzyme [23], [24]
3	Superoxide dismutase	Enzyme [25]
4	Nitrosocyanin Family	Enzyme [26]
5	Plastocyanin Family	Enzyme [18]
6	Cu-containing nitrite reductase	Enzyme [27]
7	Cu amine oxidase	Enzyme [28]
8	Particulate methane monooxygenase (pMMO)	Enzyme [29]
9	CotA	Enzyme [30]
10	Tyrosinase (melc2)	Enzyme [31]
11	PacS, CopA (*E. coli*), CopB (*E. hirae*)	Efflux system [13], [15], [16], [18], [19], [32]
12	CutC	Efflux system [17], [33], [34]
13	CopB	Efflux system [13], [34]
14	PcoA (multicopper oxidase)	Efflux system [13], [32]
15	CueO (multicopper oxidase)	Efflux system [13], [32], [34]
16	PcoC	Efflux system [2], [4]
17	CusCFBA	Efflux system [2], [4], [14]
18	PcoE	Efflux system [2], [4]
19	CtaA, CopA (*E. hirae*)	Importer [13], [16], [19], [20]
20	CopZ, Atx1 (*Synechocystis* PCC 6803)	Chaperone [16], [17]
21	CopC	Chaperone, possibly efflux [14], [17], [35]
22	CopD	Not clear, possibly importer [17], [35], [36]

**Figure 1:**
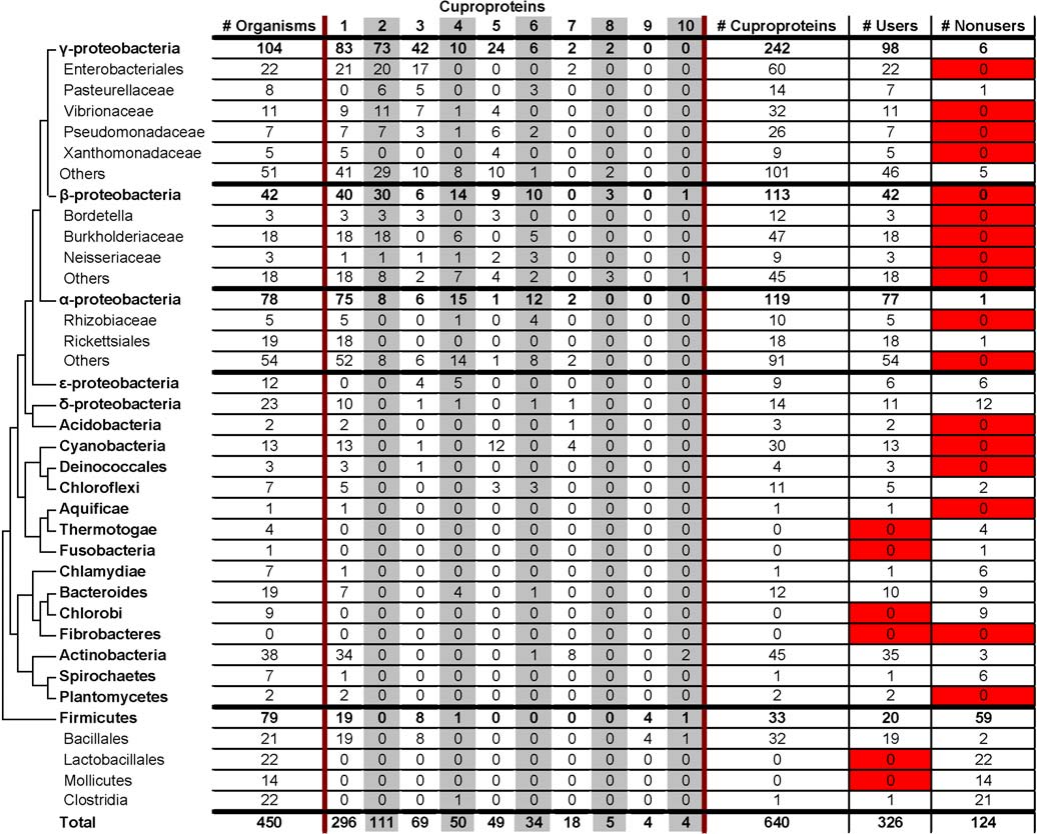
Occurrence of cuproproteins in bacteria. Phylogenetic tree was adapted from [37]. Reported is the total number of bacteria for each phylum and the numbers of bacteria that utilize a given cuproprotein. Numbers across the top refer to the cuproproteins in Table 1. The last two columns (Users and Nonusers) refer to the number of organisms in the specific phylum that are users and nonusers, respectively. Fields colored in red represent phyla where all bacteria belonging to that phylum were classified as either users or nonusers. The “# Cuproproteins” column shows the total number of cuproproteins utilized by all bacteria belonging to a particular phylum.

The numbers in the first column are the numbers used in Figures 1–3 to identify the cuproproteins.

Of 22 examined bacterial phyla represented by at least 4 completely sequenced genomes, 7 (enterobacteriales, vibrionaceae, pseudomonadaceae, xanthomonadaceae, burkholderiaceae, rhizobiaceae and cyanobacteria) consisted of bacteria that were all users. In addition, almost all bacteria from an additional 5 phyla (pasteurellaceae, rickettsiales, chloroflexi, actinobacteria, and bacillales) were users (Figure 1).

Only a few bacterial phyla (thermotogae, chlorobi, lactobacillales, and mollicutes) consisted exclusively of apparent nonusers. In addition, all but one bacterium from each of an additional three phyla (chlamydiae, spirochaetes, and clostridia) appeared to be nonusers. The phyla with the highest number of apparent nonusers were subphyla of firmicutes, which are among the most ancient organisms. Interestingly, even though we found no connection between genome size and copper use in the bacterial domain, for firmicutes, users had an average genome size of 3.93 Mb while the genome size of nonusers was only 2.27 Mb.

In archaea, the trend was somewhat reversed. There were 5 phyla with 4 or more representative organisms and only 2 of these, sulfobales and halobacteriales, consisted of exclusively users. Archaea from sulfobales prefer a harsh environment (temperature of 74°C or higher and pH of 3 or lower). In contrast, halobacteriales prefer a mild environment (temperature of 25°C and neutral pH).

**Figure 2 pone-0001378-g002:**
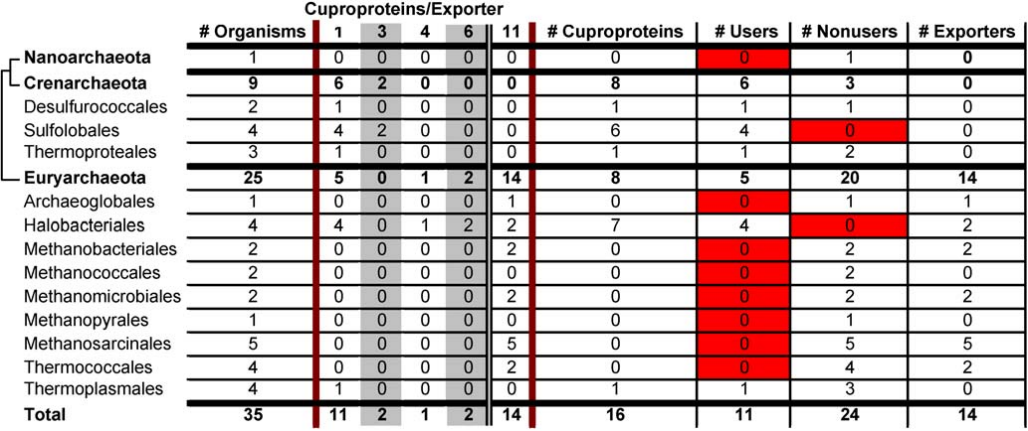
Occurrence of cuproproteins in Archaea. Reported is the occurrence (by phylum) of cuproproteins and Cu exporters in archaea. No importers, repressors, or chaperones were identified in archaea. See legend to Figure 1 for further details.

Lastly, archaea from two additional phyla (methanosarcinales and thermococcales) all appeared to be nonusers, and all but one archaeon (*Picrophilus torridus* is the exception) from the final remaining phylum, thermoplasmales, were classified as nonusers (Figure 2).

### Distribution of Cu transporter systems

**Figure 3:**
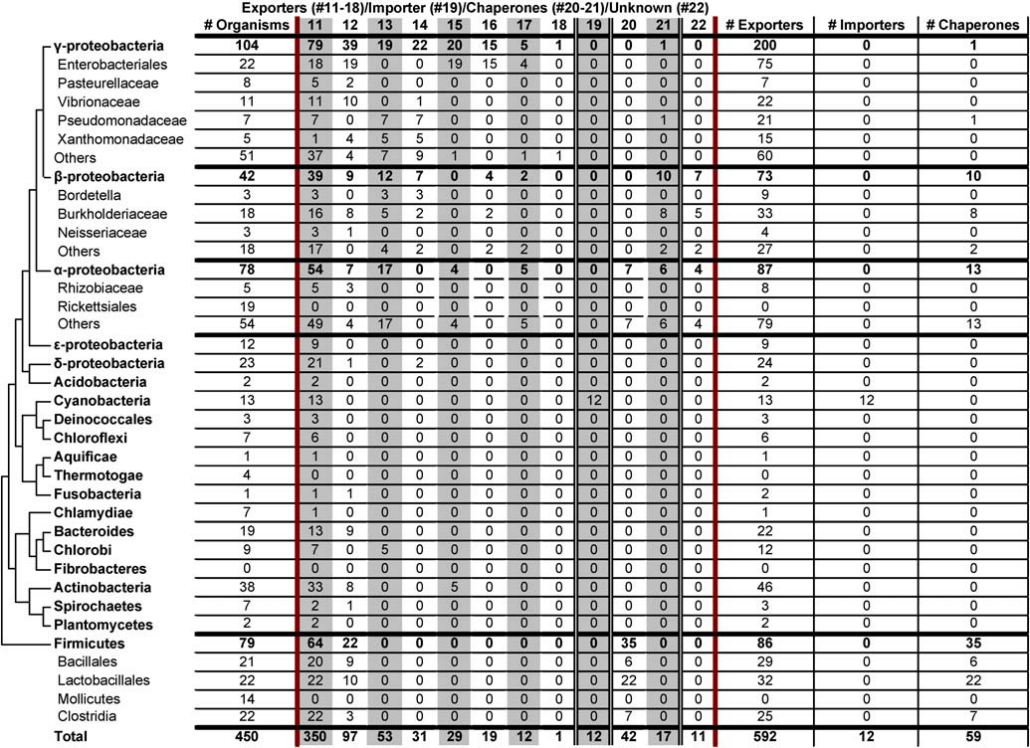
Occurrence of transporters, repressors and chaperones in bacteria. Phylogenetic tree was adapted from [37]. Reported are the number of bacteria (by phylum) which utilized a given transporter, repressor, or chaperone. Numbers across the top refer to specific transporters or chaperones (see Table 1 for protein names). Columns 10–17 are exporters, column 18 is the sole importer, columns 19–20 are chaperones, and column 21 is a transporter whose exact function has not been characterized. The last four columns report the number of exporters, importers, and chaperones present in each phyla.

Table 1 also shows all of the Cu importers/exporters analyzed in our study. CtaA (CopA in *Enterococcus hirae* [16]), the only identified Cu-specific importer, was only present in cyanobacteria. 13 different cyanobacteria were included in this study and all but one had CtaA (Figure 3). No archaea that utilize CtaA were identified (Figure 2). Each of the organisms that utilize CtaA also utilizes at least one cuproprotein.

In bacteria, PacS (CopA in *Escherichia coli* [32] or CopB in *Enterococcus hirae* [16]) was the most widespread copper exporter. PacS was identified in 355 of 450 bacteria and was present in 29 of 32 phyla (absent in mollicutes, thermotogae, and rickettsiales). In archaea, CopA (PacS) was the only transporter identified (Figure 2). It was present in 6 of 13 phyla (archaeoglobales, halobacteriales, methanobacteriales, methanomicrobiales, methanosarcinales, and thermococcales).

These two transporters, CtaA and PacS, have complimentary roles. CtaA is responsible for copper import from the periplasm to the cytoplasm and PacS is responsible for copper transport to the thylakoid [13], [18], [19]. Consistent with this, all of the examined organisms that appeared to utilize CtaA also utilize PacS.

We also examined the genomes for the other 7 copper exporters and observed some interesting features. Most organisms had copper exporters, even if they appeared to be nonusers. Nearly 50% of nonusers had copper exporters while about 25% of users appeared to lack these proteins. It is interesting to note that more than half of the bacteria that did not have exporters (79 of 142, or 56%) were parasites (overall, only 29% of the examined bacteria were parasites). Regarding the occurrence of exporters in archaea, we again observed a reversal of the trend compared to bacteria. Only 2 users (18%) had exporters while exactly half the nonusers had these proteins.

There did not appear to be any trend of importers and exporters co-occurring except for CtaA and PacS; however, PacS is nearly ubiquitous while CtaA is utilized by only a few organisms. CtaA and PacS as well as other importers and exporters are likely differentially regulated.

### Composition of cuproproteomes

For further discussion, we introduce and define the term cuproproteome. The cuproproteome of an organism is the set of proteins from its proteome which require a copper ion for their biological function (typically catalysis). Transporters, chaperones, and storage proteins constitute cellular Cu transport and maintenance machinery and are not part of the cuproproteome. Analysis of cuproproteomes revealed that organisms having cuproproteomes of identical size do not necessarily have the same set of cuproproteins. The biological function of copper is maintained by cuproproteomes, whereas the Cu transport and maintenance machinery serves an intermediate role and is not preserved during evolution when an organism lacks cuproproteins.

Distribution of cuproproteins is reported in Figures 1 and 2 for bacteria and archaea, respectively. In bacteria, the most frequently utilized cuproprotein was cytochrome c oxidase (COX). COX was identified in 296 bacteria, or 91% of the users. Moreover, among those that appeared to utilize a single cuproprotein, about 70% (94 out of 136 organisms) only utilized COX. The same was true for archaea. COX was utilized by all 11 of the archaeal users. 7 of the 11 (64%) archaeal users only utilized COX.

No other cuproproteins were nearly as widespread in bacteria. The second most frequently utilized cuproprotein was NADH dehydrogenase-2 (ND2) – utilized by 111 bacteria, or 34% of the users. Superoxide dismutase 1 (SOD1) was the only other cuproprotein utilized by more than 20% of the users (utilized by 21%), and was found predominantly in gram-negative bacteria. The remaining cuproproteins were utilized by 15% or less of the users and their frequencies were as follows:

- nitrosocyanin, 15%
- plastocyanin, 15%
- Cu-containing nitrite reductase, 10%
- Cu-amine oxidase, 6%
- pMMO, 2%
- CotA, 1%
- tyrosinase, 1%

Only four cuproproteins were identified in archaea: COX, SOD1, nitrosocyanin, and Cu-containing nitrite reductase. As previously mentioned, COX was utilized by 11 archaea (all of the users) and SOD1, Cu-containing nitrite reductase, and nitrosocyanin, were only utilized by 2 (14% of the users), 2 (14% of the users), and 1 (7% of the users) archaea, respectively.

**Figure 4:**
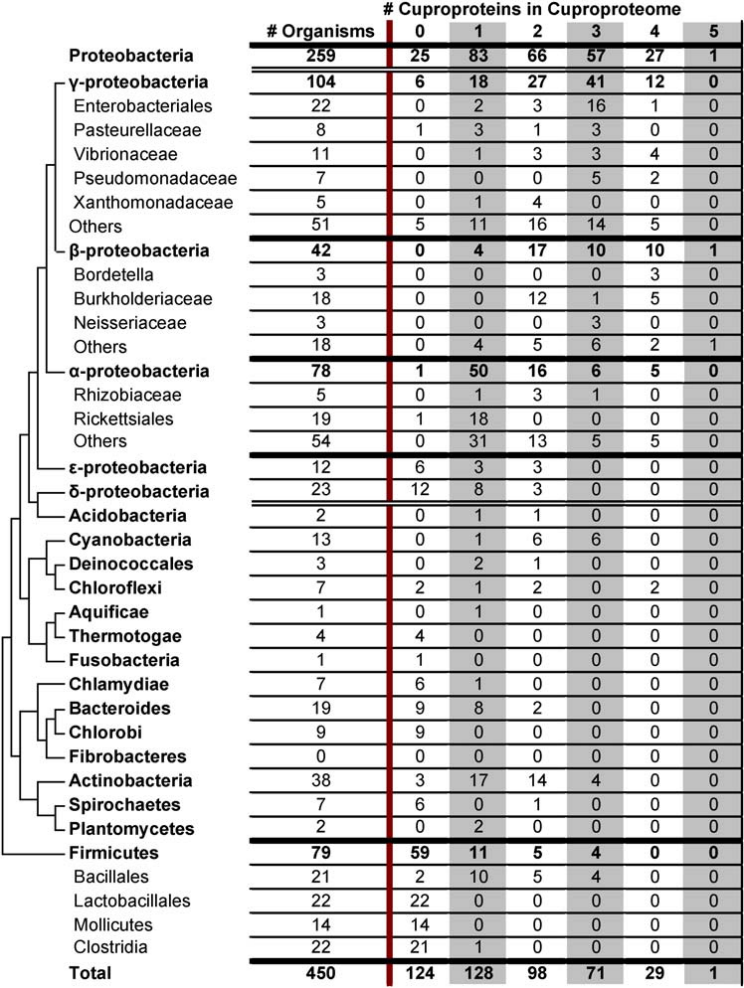
Bacterial cuproproteomes. Phylogenetic tree adapted from [37]. Numbers across the top (1–5) show the size of cuproproteomes (i.e., the number of cuproproteins in a phylum or organism). Displayed is the number of organisms from each phylum with the cuproproteome of the particular size.

Although 9 cuproproteins are known, none of the examined organisms utilized all of them. In fact, cuproproteomes of most bacteria were quite small, with the largest identified bacterial cuproproteome having only 5 cuproproteins. Only a single bacterium (*Acidovorax avenae*), less than 1% of all the bacteria included in the study, had a cuproproteome consisting of 5 cuproproteins. More common, although still infrequent, were bacteria having cuproproteomes of 4 cuproproteins; 29 bacteria or 6% had 4 cuproproteins in their cuproproteomes. The most common cuproproteomes had only one cuproprotein (128, or 28%, of all bacteria). It is interesting to note that the second most common cuproproteome size was 0 cuproproteins (124, or 28% of bacteria). So, while copper use was widespread in bacteria, most copper-dependent organisms utilized few cuproproteins (Figure 4).

**Figure 5:**
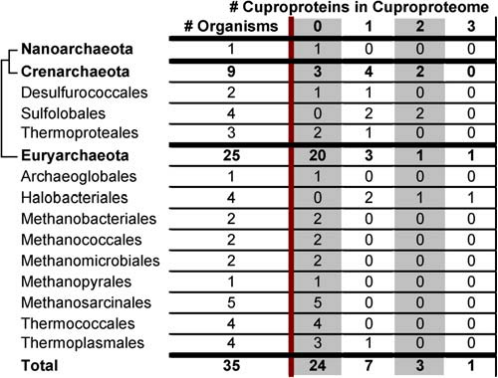
Archaeal cuproproteomes. See the legend to Figure 4.

Archaeal cuproproteomes appeared to be even smaller. The largest identified cuproproteome had only 3 cuproproteins (found only in *Haloarcula marismortui*). 3 archaea (*Sulfolobus acidocaldarius*, *Sulfolobus solfataricus*, and *Natronomonas pharaonis*), or 9%, had cuproproteomes consisting of two cuproproteins. Most archaea (∼70%) appeared to utilize no cuproproteins (Figures 2 and 5).

### Copper utilization in relation to environmental factors and organism features

We analyzed the following environmental factors and organism features to determine their effect on copper utilization:

- Habitat
- Oxygen requirement
- Genome size
- GC % in genome
- Gram strain (bacteria)
- Methanogen (archaea)
- Optimal temperature
- Optimal pH

**Figure 6:**
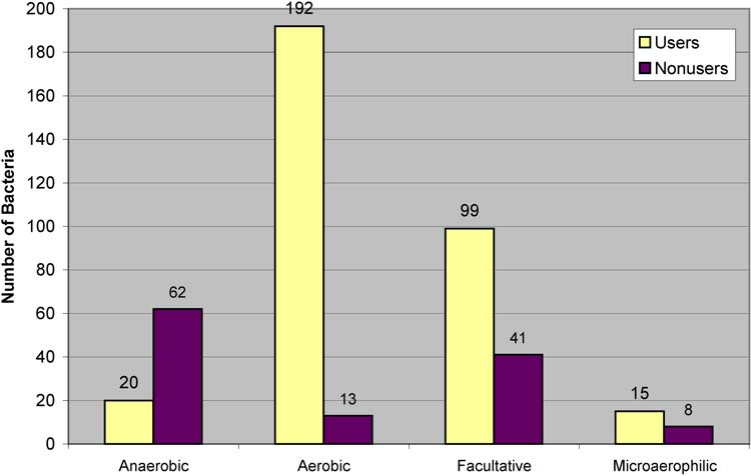
Occurrence of Cu users and nonusers among bacteria differing in their dependence on oxygen. Bacteria were divided based on their oxygen requirement into anaerobic, aerobic, facultative, and microaerophilic. Shown are the numbers of users/nonusers for each of these groups of organisms.

One feature that showed a clear association was oxygen requirement. In our study, 82 (18%) bacteria were anaerobic, 205 (46%) aerobic, 140 (31%) facultative, and 23 (5%) microaerophilic. Most anaerobic bacteria (62 of 85, or 73%) were nonusers, while most aerobic bacteria (192 of 205, or 94%) were users. 99 of 140 facultative bacteria (71%) and 15 of 23 microaerophilic bacteria (65%) were users (Figure 6). Excluding facultative organisms, a Chi-square test showed a statistically significant difference in Cu utilization between aerobic (including microaerophilic) and anaerobic organisms (P-value<0.01).

**Figure 7:**
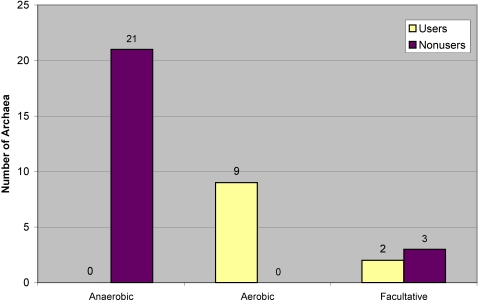
Occurrence of Cu users and nonusers among archaea differing in their dependence on oxygen. Archaea were divided based on their oxygen requirement into anaerobic, aerobic, facultative, and microaerophilic. Shown are the numbers of users/nonusers for each of these groups of organisms.

This trend was even more pronounced in archaea (Figure 7). We examined 21 anaerobic, 9 aerobic, and 5 facultative archaea. All 21 anaerobic archaea were nonusers and all 9 aerobic archaea were users. Similarly, a Chi-square test suggested a significant difference in Cu utilization between aerobic and anaerobic species (P-value<0.01). Thus, such a striking preference for the use of copper in aerobic organisms is present in both kingdoms of prokaryotes.

Not surprising, based on these results, aerobic bacteria had the largest average cuproproteomes with an average size of 1.8 cuproproteins, compared to anaerobic bacteria which had the smallest average cuproproteomes (0.4 cuproproteins). Surprising, however, is that among users, facultative and microaerophilic bacteria had larger average cuproproteomes (2.26 cuproproteins) than aerobic users which had an average cuproproteome of 1.93 cuproproteins. In archaea, where all the aerobic organisms are users and all the anaerobic organisms are nonusers, aerobic organisms clearly have the larger average cuproproteomes.

## Discussion

Oxygen levels are thought to have been quite low until 2.45 billion years ago, but by 2.32 billion years ago had sharply risen [38]. Oxygen levels rose following the origin of oxygenic photosynthetic organisms [39]. At the same time, levels of bioavailable copper increased [40]. Prior to ocean oxygenation, copper likely existed as a sulfide and was insoluble in aqueous solutions. It was suggested [41] that copper was largely unavailable for cellular life; however, upon the rise of oxygen in the atmosphere, copper was converted, at least in part to Cu^2+^, and as such, became more soluble and more readily available for biological use [41]. It is possible that at least some bacteria evolved near hydrothermal vents in waters replete with heavy metal ions, where sulfides would likely have been more soluble. However, the large-scale evolution of cuproproteins likely occurred in oxygen-rich environments where copper was plentiful. Our analyses of copper utilizing prokaryotes clearly support this idea. We found that in most prokaryotes that require oxygen are copper users, while most anaerobic prokaryotes are nonusers.

Prior to the evolution of cuproproteins, organisms may have already utilized copper exporters [16], but with the increase in soluble copper, this function became even more important as a means of removal of this metal ion. The presence of copper exporters in nonusers and the sheer number of known copper exporters suggest that copper can be taken up accidentally and these organisms need a defense mechanism against copper ions that inadvertently enter the cell.

Although exporters have been readily identified, importers have almost completely eluded researchers. As pointed out above, only one copper-dependent importer has been identified in prokaryotes. However, it is noteworthy that at least one example of an ABC transporter has been reported which has broad specificity for a number of metal ions, including copper, and this could potentially explain copper uptake by prokaryotes [42].

Additionally, while there are examples of cuproproteins that obtain copper from the cytoplasm (for example, plastocyanin gets copper from PacS, which exports copper from the cytoplasm to the thylakoid [18], [19]) most cuproproteins are periplasmic or membrane proteins and therefore it is possible that little cytoplasmic copper is needed for these proteins, which could help explain the lack of copper-specific importers.

In this study we only included proteins that are unable to function without copper. While no reports of prokaryotic enzymes evolving by changing the catalytic copper to other metals exist, it is a possibility, although unlikely, that some of the proteins we have identified as cuproproteins actually utilize a different metal ion.

Various cuproproteins have been identified by researchers over the years. The two most widespread cuproproteins are COX and ND2, both of which are involved in the electron transport chain. The redox properties of copper make this metal ion a prime candidate for use in the active sites of oxidoreductases involved in energy generation. In fact, in bacteria, 84% of the cuproproteins, including COX and ND2, are involved in energy producing pathways, and in archaea 86% of the cuproproteins are involved in ATP production. These data suggest that ATP generation via oxygen-dependent respiration is the major use of copper in biology. Being readily available in the environment and with such wide use in oxygenic organisms, it is a paradox that the utilization of this metal is restricted to only a few enzymes in prokaryotes.

Copper use in organisms does have its associated dangers though, and organisms which have evolved to use this metal have to have ways to deal with copper toxicity. Copper toxicity comes from its ability to produce reactive oxygen species (ROS). Thus, copper is intimately involved in the cellular control of redox homeostasis. SODs are important proteins which protect the cell against ROS by removing superoxide anions. Each of the known SODs requires at least one metal cofactor. Among these is a cuproprotein, SOD1, which utilizes both copper and zinc. Other SODs are known to use iron, manganese, or nickel [25], [43]. Interestingly, copper is used in both respiration and the associated removal of ROS, which are by-products of oxygen-dependent respiration.

Another interesting cuproprotein is a multicopper oxidase, CotA, which is a spore coat protein. Only four bacteria (and no archaea) were found to utilize CotA, all of which were Bacillales. It appears that CotA is a recently evolved cuproprotein. Other multicopper oxidases studied (CueO and PcoA) are components of efflux systems.

Cuproproteomes appear to be quite small in prokaryotes. In both bacteria and archaea, the most common cuproproteome size for users was one, and frequently this single cuproprotein was COX. Interestingly, organisms with cuproproteomes of the same size do not necessarily utilize the same cuproproteins, and most cuproproteins are used in a limited number of organisms.

The use of copper can be compared (or rather contrasted) with that of another trace element, selenium. The size of prokaryotic selenoproteomes is highly variable, from 0 to 56 [10], [44]. However, approximately 80% of prokaryotes do not have selenoproteins. Thus, whereas prokaryotic selenium use shows a mosaic pattern and most selenoprotein-rich organisms are anaerobic, copper is utilized by a limited number of processes (essentially restricted to respiration), but in many aerobic organisms.

Our study was based on the premise that most cuproproteins are already known. The striking occurrence of these proteins in aerobic organisms suggests that we could reliably identify most users. The possibility cannot be excluded, however, that additional, unknown Cu-dependent proteins are present in some prokaryotes. Nevertheless, while additional cuproproteins would increase the size of cuproproteomes, it is unlikely that this would lead to identification of many additional users.

It would be interesting to expand this work to the third super-kingdom, eukaryotes. By definition, these organisms evolved in oxygenated environments, but whether copper utilization remained widespread during evolution of eukaryotes is not known. There is some overlap between eukaryotes and prokaryotes on which a study of this nature could be based; for example, both prokaryotes and eukaryotes have COX and SOD1.

Finally, we have pointed out that some very important enzymes require a copper cofactor, and while many of them are periplasmic and membrane-bound, some are intracellular. In the future, it would be interesting to examine the means by which prokaryotes bring copper into the cell under conditions of deficiency in this trace element. Very little is known about copper import in prokaryotes; in many cases unspecific import systems would likely be sufficient, but whether this is the major route for copper use is not known.

## Materials and Methods

### Genomic sequence resources

In our study, we examined fully sequenced prokaryotic genomes (450 unique bacterial and 35 unique archaeal genomes were available as of January 31, 2007). Only a single strain of each species was included in this research. A list of fully sequenced prokaryotic genomes, both those used in this research and those completed since we began our research, can be found on the NCBI website at: http://www.ncbi.nlm.nih.gov/sutils/genom_table.cgi.

### Identification of Cu-utilizing organisms, Cu-specific transporters, and cuproproteins

Initially, primary literature was used to identify Cu-dependent proteins, and Cu importers, exporters, and regulators. Cuproproteins were included in the set if their close homologs were experimentally demonstrated to be specific for copper. Copper transporters were included if they were previously reported to be copper-specific or if they had a much higher affinity for copper than other metal ions. A complete list of identified proteins, importers, exporters, and regulators is shown in Table 1.

Organisms that had at least one copper-dependent protein were considered copper-dependent, or users. If we were unable to find at least one cuproprotein in an organism it was designated as copper-independent or a nonuser. Determining if a specific organism had cuproproteins was accomplished in the following steps:

- Using primary literature, organisms were identified that had a particular cuproprotein;
- TBlastn [45] search with default parameters was performed against all organisms in the study using the protein sequences from the step above;
- The organism from each phylum having a proteome containing the examined cuproprotein and having the highest homology to the query sequence was identified;
- A new Blast search was performed using the protein sequence from the previous step as query sequence and only searching the genomes from that particular phylum. In the case that no organism from the phylum had the original protein, this additional search was not performed;
- Manual analysis of the resulting sequences to determine which organisms had the cuproprotein in question.

### Other analysis tools

Inferred phylogenies were used to verify similarity to putative cuproprotein sequences. Multiple sequence alignments of proteins and inferred phylogenies were created using ClustalW [46] with default parameters. Manual analysis of the inferred phylogeny was performed to identify which candidate sequences clustered around the putative cuproprotein.

### Identification of environmental and other factors for each organism

To examine trends of copper utilization we collected information for various environmental factors (e.g., habitat, oxygen requirement, optimal temperature and optimal pH), and other factors and properties (e.g., genome size, GC content and Gram strain) which were retrieved from the NCBI prokaryotic genome project database (http://www.ncbi.nlm.nih.gov/sites/entrezcmdFiledbgenomeprj) for all examined archaeal and bacterial species.

## Supporting Information

Table S1 (0.18 MB XLS)
